# Case Report: Early Distant Metastatic Inflammatory Myofibroblastic Tumor Harboring *EML4-ALK* Fusion Gene: Study of Two Typical Cases and Review of Literature

**DOI:** 10.3389/fmed.2022.826705

**Published:** 2022-02-24

**Authors:** Qianqian Han, Xin He, Lijuan Cui, Yan Qiu, Yuli Li, Huijiao Chen, Hongying Zhang

**Affiliations:** ^1^Department of Pathology, West China Hospital, Sichuan University, Chengdu, China; ^2^Department of Pathology, Suining Central Hospital, Suining, China

**Keywords:** inflammatory myofibroblastic tumor, early distant metastasis, *EML4-ALK* fusion variant 3a/b, NOTCH1, ARAF, ALK inhibitor

## Abstract

Inflammatory myofibroblastic tumor (IMT) is a distinctive neoplasm that frequently arises in the lung and accounts for ~1% of lung tumors. Distant metastatic IMT is extremely rare and has been poorly investigated. This analysis was specifically performed to explore the clinicopathological and genetic features of early distant metastatic IMT. Two typical patients with distant metastatic IMTs were selected, which accounted for 1.13% of all diagnosed IMTs in the last 5 years. One patient was a 55 year-old male, and the other patient was a 56 year-old female. Both primary tumors arose from the lung, and the initial clinical symptoms of the two patients involved coughing. Both of the imaging examinations showed low-density nodular shadows in the lungs with enhancement around the mass. Microscopically, dense arranged tumor cells, prominent cellular atypia, and high mitotic activity with atypical form were more prominent in the metastatic lesions than in the primary lesions. All of the primary and metastatic tumors in both cases showed positive anaplastic lymphoma kinase (*ALK*) immunostaining and *ALK* rearrangement *via* fluorescence *in situ* hybridization. The *EML4* (exon 6)*-ALK* (exon 20) fusion variant (v3a/b) was identified by using next-generation sequencing (NGS) and was verified by using reverse transcription polymerase chain reaction (RT-PCR). Furthermore, intronic variants of *NOTCH1* and synonymous variants of *ARAF* were also detected *via* NGS in one IMT for the first time and were verified in all of the primary and metastatic lesions *via* PCR. Distant metastasis occurred during a short period of time (1 and 2 months) after the first surgery. One patient presented with multiple metastases to the subcutaneous tissue and bone that responded to *ALK* inhibitor alectinib therapy, and the tumor was observed to regress 10 months after the initial *ALK* inhibitor therapy. In contrast, the other patient presented with subcutaneous neck metastasis without *ALK* inhibitor treatment and succumbed to the disease within 3 months after the surgery. This study demonstrated the possible role of *EML4*-*ALK*v3a/b in the malignant progression of IMT and proposed certain therapeutic effects of *ALK* inhibitors on multiple metastatic IMTs.

## Introduction

Inflammatory myofibroblastic tumor (IMT) is a distinctive neoplasm composed of myofibroblastic and fibroblastic spindle cells accompanied by chronic inflammatory infiltration (1). IMTs frequently affect the lung, mesentery, omentum, and retroperitoneum, and most commonly occur in children and young adults (2, 3). However, among lung tumors, these entities are extremely rare and account for ~1% of adult lung tumors (4). Approximately 50–60% of IMTs harbor anaplastic lymphoma kinase (*ALK*) gene rearrangement, which can fuse with multiple partner genes, including *ATIC, CLTC, EML4, NPM, TFG, TPM3/4*, and other genes (5, 6). In *ALK*-negative IMTs, *ROS1*, and *NTRK3* gene rearrangements are the most frequent mutations and exist in 5–10% of all IMTs, whereas *RET* and *PDGFRB* rearrangements are found in a few IMTs (7, 8). IMT is a borderline tumor with an appropriate prognosis; the metastatic rate is extremely low (<5%) (1). The clinicopathological and molecular characteristics of distant metastatic IMTs are largely unknown.

To the best of our knowledge, approximately 50 metastatic IMTs have been reported in the English literature (9–16), and most metastatic sites involve the lung, liver, and brain. Thirty-seven patients underwent ALK immunostaining, and 19 patients demonstrated positive results. Gene detection was only performed in 10 patients, and 8 patients had ALK rearrangements, including 3 patients with *EML4-ALK* fusions (8, 14–21). Although several studies have demonstrated *EML4-ALK* fusions in up to 20% of *ALK*-rearranged IMTs (8), the association between different *EML4-ALK* fusion variants and IMT disease progression has been insufficiently investigated. Until now, pathological indicators for predicting the biological behavior and prognosis of IMT have been deficient. Herein, we present two cases of metastatic IMTs, harboring *EML4-ALK* fusion, and review the literature to summarize the clinicopathological and genetic features that may help to predict the tendency of metastasis in IMT.

## Case Presentation

### Clinical Characteristics

**Figure 1:**
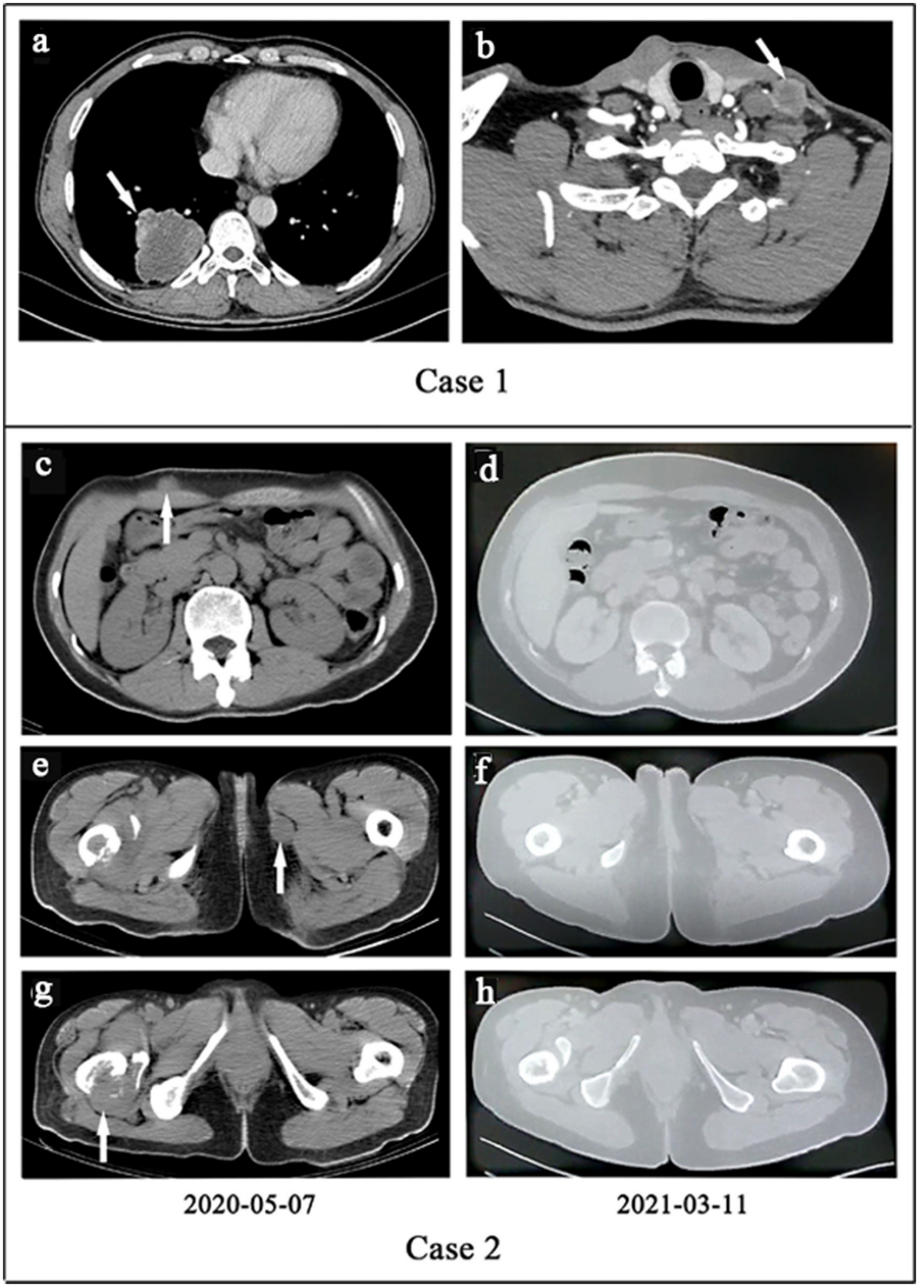
**(a,b)** Case 1. **(a)** The computed tomography (CT) of primary lesion revealed a large, irregular mass with a non-uniform density shadow attached to the lower lobe of the right lung. **(b)** The CT of metastatic lesion showed an uneven density lesion within subcutaneous of the left neck with an unclear border. **(c–h)**. Case 2. Treatment efficacy with Alectinib in Case 2 based on CT scans. **(c,e,g)** Before Alectinib treatment. Multiple low-density nodules were observed in subcutaneous of the right upper abdomen, the inner side of the left thigh, and the right femur. **(d,f,h)** Ten months after Alectinib treatment, multiple soft tissue density nodules decreased or disappeared.

Case 1 was a 55 year-old male, and case 2 was a 56 year-old female. Both of the patients had been coughing for 2 and 6 months before surgical resection. The imaging features are shown in Figure 1. Computed tomography (CT) scans found 4-cm low-density nodular shadows with slight enhancement around the mass in the right inferior lobe (Figure 1a) and a 3.3-cm lung nodule in the right upper lobe. In case 1, the metastatic lesion of the left neck was found and grew rapidly to 3 cm 1 month after the initial surgery, and it was also resected (Figure 1b). Case 2 had undergone lung lesion resection in December 2019. Nevertheless, subcutaneous painless lesions in the right thigh and the iliac region were found 2 months after the operation and were resected in March 2020. The resection margins were negative for all five tumor lesions. Detailed clinical features are summarized in Supplementary Table 1.

### Pathological Features

**Figure 2:**
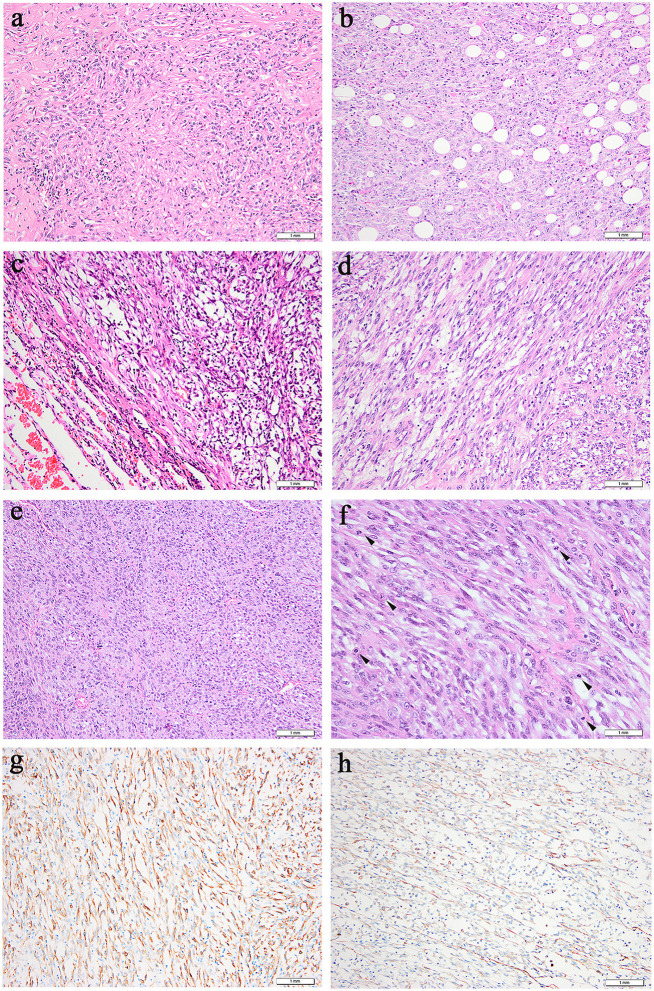
Microscopic features (hematoxylin and eosin). **(a,b)** Case 1, **(c–f)** Case 2. **(a)** Prominent collagenous background was observed in the primary lung lesion. (**b)** Metastatic neck lesion showed adipose infiltration. **(c)** A clear boundary between tumors and normal lung tissue in some areas could be observed in primary lung lesion. **(d)** Spindle tumor cells of thigh lesion arranged in fascicular. **(e)** Metastatic lesion of ilium exhibited densely arranged tumor cells. **(f)** More dense tumor cells arrangement, more obvious atypia, more vacuolated and prominent nucleoli, higher mitotic activity (an arrowhead is shown) were observed in metastatic lesion of ilium. **(g)** Positive cytoplasmic ALK staining was observed in all 5 specimens. (**h)** Focally positive staining for smooth muscle actin was shown in Case 2.

All primary and metastatic lesions (5 specimens) were presented as gray and white nodular masses with solid and medium quality. Hematoxylin and eosin staining (H&E) was performed on all of the primary and metastatic lesions (Figure 2). Microscopically, tumors were arranged in fascicles and composed of spindle-shaped cells with lymphoplasmacyte and/or eosinophil infiltration. The nucleus was round or oval with a prominent nucleolus, and tumors of metastatic focus were homologous to those of the primary focus. Compared with primary tumors, metastatic tumors exhibited more densely arranged tumor cells, more obvious cellular atypia, more vacuolated nuclei, more prominent nucleoli, and higher mitotic figures with atypical forms (3–4/2 mm^2^ in the lung and 10–15/2 mm^2^ in the neck of Case 1; 8–9/2 mm^2^ in the lung, 30–40/2 mm^2^ in the thigh, and 20–30/2 mm^2^ in the ilium of Case 2. Many new thin-walled capillaries were arranged in the tumors with diffuse lymphoplasmacyte infiltration. Some cellulose-like necrosis was observed in Case 1.

### Immunohistochemistry Profile

All of the specimens showed a similar expression of immunostaining. Specifically, ALK-IHC showed diffuse cytoplasmic reactivity in all 5 specimens (Figure 2g), and the density in the metastatic lesions was higher than that in the primary lesions. Focal staining for smooth muscle actin was observed in Case 2 (Figure 2h) but was negative in Case 1. No tumor cells demonstrated positive staining with anti-S-100 protein, CD34, cytokeratin, or desmin.

### Molecular Analysis

**Figure 3:**
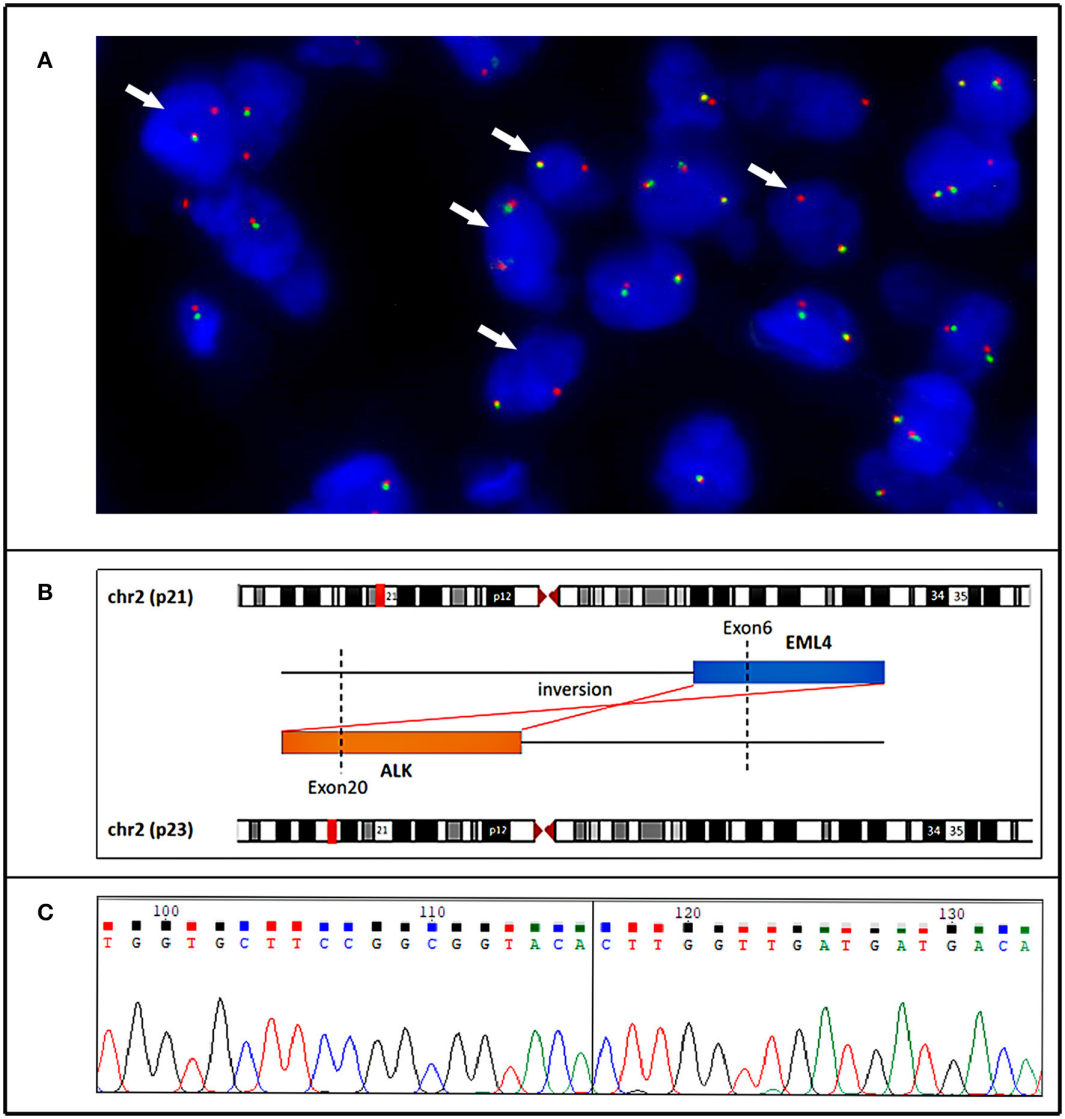
Case 1. **(A)** Fluorescence *in situ* hybridization demonstrating an unbalanced rearrangement of the *ALK* locus in the neoplastic cells (loss of a green signal with an extra red signal). **(B)** Next generation sequencing (NGS-) based technology showing detection of *ALK* (exon 20) and *EML4* (exon 6) fusion and breakpoint information between the two genes. **(C)** A partial nucleotide sequence of the *EML4-ALK* fusion transcript, and the fusion site (arrow) involved exon 20 of the *ALK* gene and exon 6 of the *EML4* gene.

**Figure 4:**
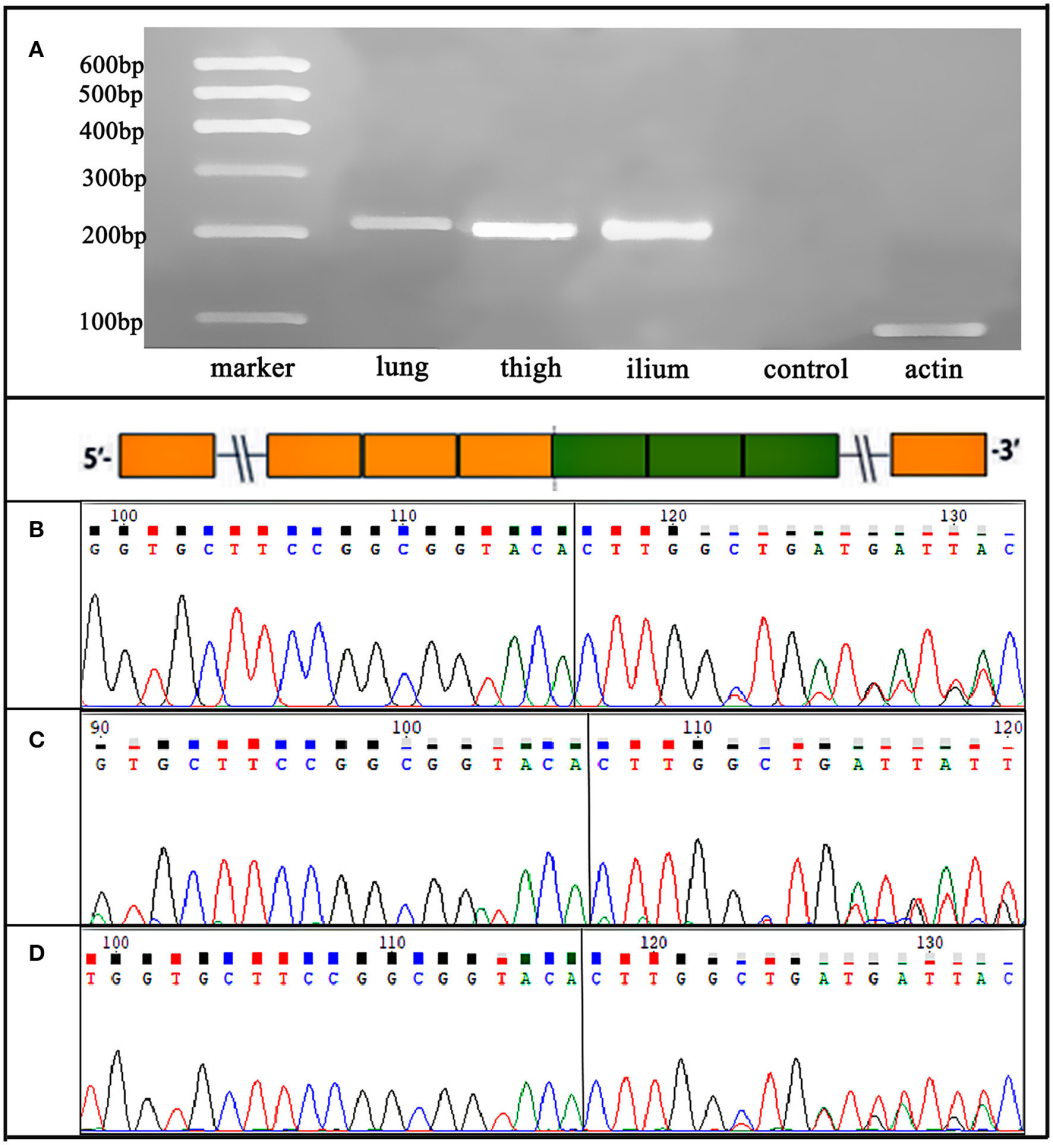
Case 2. **(A)** RT-PCR for lung, right thigh, and ilium lesions confirmed an *EML4-ALK* fusion variants, which were detected by NGS. **(B-D)** Sanger sequencing verified the sequence of the *EML4* (exon 6)*-ALK* (exon 20) fusion transcript in the three primary and metastatic lesions.

All five specimens were detected by using next-generation DNA sequencing (NGS) and were verified by using reverse transcription polymerase chain reaction (RT–PCR) or PCR. *ALK* rearrangement was confirmed *via* fluorescence *in situ* hybridization (FISH) in the pulmonary lesion of Case 1 (Figure 3A) and in all three lesions of Case 2. Subsequent NGS broad molecular profiling of the tumor tissue was performed, and the fusion break-point involved exon 6 of *EML4* and exon 20 of *ALK* in all of the primary and metastatic tumors of these cases with identical fusion sequences. Confirmatory RT-PCR and subsequent Sanger sequencing confirmed the fusion of *EML4-ALK* in all of the tumors (Figures 3, 4). Otherwise, intronic variants of *NOTCH1* in exons 2 and 24 and synonymous variants of *ARAF* in exon 4 were also detected by using NGS in the above three primary and metastatic lesions of Case 2. PCR was performed, and *NOTCH1* and *ARAF* variants were demonstrated *via* Sanger sequencing (Supplementary Figure 1).

### Treatment and Follow-Up

Patient one succumbed to disease only 3 months after initial presentation. In May 2020, whole-body CT examinations showed many soft tissue density lesions in the left axillary subcutaneous area, right upper abdominal subcutaneous area, left thigh medial subcutaneous area, and right iliac fossa area. Hence, the patient was orally administered alectinib (600 mg, two times daily). Whole-body CT in March 2021 indicated that all of the tumors had significantly responded (Figures 1c–h). Currently, the patient is still in good clinical condition without adverse events 16 months after the initiation of alectinib treatment.

## Discussion

The incidence of distant metastatic IMT was very low, accounting for 1.13% of the 177 identified patients with IMT for investigation in our institution from January 2016 to July 2021. A search of the English literature indicated that approximately 50 metastatic IMTs from some isolated cases or portions of other series studies had been described thus far (Supplementary Table 2) (9–16). Furthermore, epithelioid IMT with malignant potential was not included in this study. Metastatic lesions involved the lung, brain, lymphonodus, bone, abdomen, and pelvis. Additionally, the patient ages ranged from 5 to 81 years (mean: 36.2 years; median: 35.5 years), with a male-to-female ratio of 1.1:1. The distribution of age throughout all of the age groups was consistent with non-metastatic IMT without a significant difference (7). However, there was a slight male predominance in the gender distribution. Primary and metastatic lesions were simultaneously found in nearly half of the IMTs (16/35, 45.7%), and other metastatic intervals ranged from 1 month to 9 years. In this study, two lung IMTs with early distant metastasis were included: one IMT metastasized to the subcutaneous neck, and one IMT had multiple systemic metastases, with intervals of 1 and 2 months.

The correlation between morphology and biological behavior in IMT is still ambiguous. The histological characteristics of 50 metastatic IMTs were analyzed, and the primary tumor size was 1.3–22 cm (median: 6.8 cm; mean: 7.2 cm). Varying degrees of cellular or nuclear atypia in primary lesions were described in 25 patients, whereas a few patients harbored high mitotic figures (most of which were 30/2 mm^2^). Among 16 previously reported metastatic IMTs of primary lung locations with detailed pathological descriptions, 10 IMTs had atypical features with large atypical nuclei, distinct nucleoli, high mitotic activity, or nuclear pleomorphism. Similar to earlier cases, some atypical morphological features were also shown in our cases, such as hypercellularity and prominent mitotic figures. Traditionally, larger tumor size, hypercellularity, the presence of tumor cell necrosis, high mitotic activity, and the presence of ganglion-like cells were considered to be associated with poor survival in IMTs (8, 9). This series further illustrated that hypercellularity, atypical spindled cells, and high mitotic activity may predict malignant potential, and pathologists should be aware of these morphological changes. Of course, a further analysis of large samples is needed.

Previously, *ALK*-negative IMTs were considered to have higher metastatic potential (1). One study stated that *ALK* reactivity may be a favorable prognostic indicator in IMT because none of their 6 metastatic IMTs were reactive for *ALK* (9). However, among the 37 previously reported metastatic IMTs with *ALK* immunostaining information, more than half of the patients (51.4%, 19/37) harbored positive results, thus suggesting that ALK immunoreactivity does not appear to correlate with metastasis (Supplementary Table 2). Among the 4 patients with detectable specific *ALK* fusion partners, 3 harbored *EML4-ALK* fusions, and the other harbored *CARS-ALK* fusions. *EML4-ALK* may affect tumorigenesis and be used to identify IMT that develops malignant transformation or recurrence (15). In our series, the ALK staining density in most areas of the metastatic lesions was higher than that of the primary lesions, thus indicating a possible role of ALK in IMT metastasis. The prognostic significance of *EML4* as a fusion partner in IMTs remains unclear, but a potential link between *EML4-ALK* fusion and a malignant clinical course should be explored in further studies with a larger series.

In this study, both primary and metastatic lesions of the two IMTs were positive for ALK and harbored *EML4* (exon 6)*-ALK* (exon 20) fusion. To date, eighteen *EML4-ALK* fusion IMTs have been reported (including the present two IMTs) (8, 13–20) (Supplementary Table 3), eleven of which are located in the lung (11/18, 61.1%). The other cases included two IMTs in the abdominopelvic cavity, two IMTs in the limbs, one IMT in the head, one IMT in the trachea, and one IMT in the hypopharynx. Seven of eighteen *EML4-ALK-*rearranged IMTs had different degrees of cellular atypia and/or mitosis. Some studies have reported that *EML4-ALK* rearrangement is often combined with epithelioid cell morphology and distant metastasis or local infiltration tendency (8, 14, 17). Among the 6 cases (6/18, 33.3%) investigated with DNA sequencing, exon 6 of *EML4* fused with exon 20 of *ALK* in 4 cases, including the two IMTs that we investigated; additionally, exon 4 of *EML4* fused with exon 20 of *ALK* in one case, and the other case involved exon 2 of *EML4* and exon 20 of *ALK*. Some clinical studies have demonstrated that the *EML4* (exon 6)-*ALK* (exon 20) fusion variant (3a/b) is a high-risk feature, conferring accelerated metastatic spread, ALK inhibitor resistance, and worsened prognosis in ALK-positive non-small-cell lung cancer (NSCLC) (22–24). The 3a/b variants of the *EML4-ALK* fusion gene were identified by using FISH and Sanger sequencing in a malignant hypopharynx IMT, and strong ALK immunoreactivity was also observed in neoplastic cells (8), which was best illustrated by our cases. Early distant metastasis occurred in our two cases, which was also combined with the fusion of *EML4-ALK* fusion variant 3a/b, thus indicating that this specific fusion variant may also play a considerable role in the malignant progression of IMT. The presence of *EML4-ALK* fusion variant 3a/b in different malignant neoplasm types demonstrated the hypothesis that identical *ALK* fusions may drive inappropriate activation of the same kinase signaling pathway and could be oncogenic in disparate cellular lineages. The determination of *ALK* fusion status should be considered as part of the initial workup for this phenomenon to select patients for more aggressive disease surveillance.

Otherwise, synonymous variants of *ARAF* and intronic variants of *NOTCH1* were detected in one case (Case 2). A few examples have indicated that synonymous mutations in diseases may act as driver mutations (25). *NOTCH* signaling was shown to physically regulate interactions between adjacent cells as an evolutionarily conserved intercellular signaling pathway. *NOTCH1* encodes a member of the *NOTCH* family of proteins and drives an oncogenic transcriptional program that promotes cell growth proliferation and survival (26). Importantly, the oncogenic effects of *NOTCH1* are closely linked to the activation of the *MYC* oncogene and can regulate overlapping transcriptional programs in a feed-forwards loop transcriptional circuitry that amplifies the oncogenic effects of *NOTCH1* (25, 27). *ARAF* belongs to the RAF subfamily of the serine/threonine kinase family and may be involved in cell growth and development. They interact with the *RAS-MAPK* pathway and act as binary molecular switches, controlling intracellular signaling pathways and participating in basic cellular processes, such as cell proliferation, differentiation, adhesion, migration, and apoptosis (28, 29). *NOTCH1* or *ARAF* mutations have not been previously reported in IMTs. In this case, the disease progressed very rapidly, multiple subcutaneous metastases occurred in the whole body within a few months, and the malignancy of the metastatic lesion was higher than that of the primary lesions. Whether *NOTCH1* and *ARAF* act together with *ALK* mutations in the progression of IMTs needs further exploration.

From a morphological perspective, the main differential diagnostic consideration is spindle cell neoplasms, especially with inflammatory infiltration. First, the occurrence of distant metastasis and moderate-to-severe atypia excludes inflammatory pseudotumors and myofibroblast proliferation. Second, sarcomatoid carcinoma usually exhibits high-grade spindle cell morphology, but negative cytokeratin expression refuted this diagnosis. Third, epithelioid inflammatory myofibroblastic sarcoma is composed of plump epithelioid or histiocytoid tumor cells with vesicular chromatin and accompanied by a unique pattern of nuclear membrane or perinuclear *ALK* immunoreactivity (30). Moreover, the molecular detection of *EML4*-*ALK* fusion excludes leiomyosarcoma and other spindle mesenchymal tumors.

For prognosis, the follow-up time was 3 weeks to 36 months (median: 9 months; mean: 11.2 months) in 30 metastatic patients with follow-up information. However, not all of the patients undergoing *ALK* inhibitor therapy had a favorable prognosis. The patients without *ALK* inhibitor therapy were followed up for 3 weeks to 24 months (median: 8.5 months; mean: 9.4 months), and the disease-free survival rate was only 40.9% (9/22). The follow-up time for the patients treated with *ALK* inhibitors was 4–36 months (median: 13.5 months; mean: 16.4 months), and the disease-free survival rate was 75% (6/8), thus proving that the usage of *ALK* inhibitors is crucial to improve the survival of the patients. Although *EML4-ALK* variant 3a/b-fused tumors may harbor a high risk of *ALK* inhibitor resistance, one of our cases responded effectively to the administration of the second-generation *ALK* inhibitor alectinib and encompassed an appropriate prognosis. Likewise, to the best of our knowledge, only one previous IMT with *EML4-ALK* fusion confirmed by FISH that responded to alectinib treatment was initially reported (15). The carcinogenic pathway is mainly mediated by *ALK*, and the fusion partner gene activates the *ALK* tyrosine kinase domain through a driver/oligomerization mechanism as a promoter (15). *EML4-ALK* fusion variant 3a/b-driven cancers were characterized by oncogene dependence, and ALK inhibitors may be more effective for this tumor subgroup (17, 18).

Of the *ALK* inhibitor treatments, crizotinib and two other ATP-competitive *ALK* inhibitors (ceritinib and alectinib) are approved for use as first-line therapies, whereas ceritinib and alectinib are two second-generation *ALK* inhibitors with acceptable safety profiles that have been proved to be effective against many of the prominent forms of crizotinib-resistant *ALK*-positive NSCLC (31). In the USA, oral alectinib monotherapy is indicated for the treatment of patients with ALK-positive metastatic NSCLC, as detected by a Food and Drug Administration-approved test (32). Alectinib was observed to inhibit autophosphorylation of *ALK* and to suppress phosphorylation of STAT3 and *ALK* (but not ERK1/2) in NSCLC cells expressing *EML4-ALK*, as well as inhibiting the growth of multiple cell lines bearing *ALK* fusions, amplifications, or activating mutations (31, 33). Two of sixteen *EML4-ALK-*rearranged IMTs (14, 16) were negative for *ALK via* IHC, which underlies the risk of negating systemic therapy with kinase inhibitors. Moreover, many *ALK* fusion variants show a diffuse cytoplasmic pattern, and the fusion partners cannot be identified *via* IHC, although a few fusions are related to the nuclear membrane (*RANBP2-ALK*), perinuclear (*RRBP1-ALK*) or a cytoplasmic granular pattern (*CLTC-ALK*) of *ALK* immunohistochemistry (1, 30). Thus, FISH or NGS detection at the DNA level should be suggested in IMTs with typical morphological features, regardless of the IHC results, especially in those patients with malignant potential.

In summary, this study presented two extremely rare early distant metastatic IMTs, both harboring *EML4-ALK* fusion variants 3a/b. We summarized the reported distant metastatic IMTs and found that tumor cell atypia and high mitotic activity may often indicate metastatic possibility. A systematic metastatic IMT was emphasized. In addition to *EML4-ALK* fusion variant 3a/b, synonymous variants of *ARAF* and intronic variants of *NOTCH1* were also detected *via* NGS, and this patient responded effectively to treatment with the second-generation *ALK* inhibitor alectinib. *EML4-ALK* fusion variants 3a/b could be oncogenic in disparate cellular lineages, thus causing the malignant progression of IMT, and *ALK* inhibitor administration could improve the prognosis. Of course, more studies are needed to clarify the relationship between *EML4-ALK* fusion variants and IMT progression, prognosis, and treatment effectiveness. Further studies using additional cases are needed to explore the prognostic factors of IMT to facilitate timely treatments.

## Data Availability Statement

All datasets generated for this study are included in the article/Supplementary Material.

## Ethics Statement

The studies involving human participants were reviewed and approved by the Ethics Committee of West China Hospital of Sichuan University. The patients/participants provided their written informed consent to participate in this study.

## Author Contributions

QH: data analysis, test performance, writing, and review of the manuscript. XH: review of the manuscript and histopathology. LC: data curation. YQ: collection of information and software. YL: molecular experiments. HC: review of histopathology. HZ: project administration, study design, and the manuscript revision. All authors contributed to the article and approved the submitted version.

## Funding

This work was supported by the National Natural Science Foundation of China (Grant No. 81972520), the 135 Project for Disciplines of Excellence-Clinical Research Incubation Project, West China Hospital, Sichuan University (Grant No. 2018HXFH011), Medical Science and Technology Project of Sichuan Provincial Health Commission (Grant No. 21PJ002), and Post-Doctor Research Project, West China Hospital, Sichuan University (Grant No. 2021HXBH004).

## Conflict of Interest

The authors declare that the research was conducted in the absence of any commercial or financial relationships that could be construed as a potential conflict of interest.

## Publisher's Note

All claims expressed in this article are solely those of the authors and do not necessarily represent those of their affiliated organizations, or those of the publisher, the editors and the reviewers. Any product that may be evaluated in this article, or claim that may be made by its manufacturer, is not guaranteed or endorsed by the publisher.
