# Supplementary material for: Case Report: Early Distant Metastatic Inflammatory Myofibroblastic Tumor Harboring EML4-ALK Fusion Gene: Study of Two Typical Cases and Review of Literature
Source: Front Med (Lausanne). 2022 Feb 24;9:826705. doi: 10.3389/fmed.2022.826705 (PMC8907662; doi:10.3389/fmed.2022.826705)
Supplement: Supplementary file 1 [file Table_1.DOCX]

**Supplemental Table 1. Clinicopathologic characteristics of the two metastatic IMTs***

| **Case number** | **Age (y)** | **Gender** | **Lesion** | **Lesion type** | **Tumor size (cm)** | **Smoke History** | **Primary symptom** | **Metastatic Interval** | **Molecular genetics** | **Other mutation** | **Treatment** | **Surgical margins** | **Follow-up** |
| --- | --- | --- | --- | --- | --- | --- | --- | --- | --- | --- | --- | --- | --- |
| 1 | 55 | M | Lung | Primary | 4 | Yes | Cough | 1mo | *EML4*(exon6)-*ALK*(exon20) | */* | Surgical resection | Neg | DOD/3mo |
|  |  |  | Neck | Metastastic | 3 |  |  |  |  |  |  | Neg |  |
| 2 | 56 | F | Lung | Primary | 4 | No | Cough | 3mo | *EML4*(exon6)-*ALK*(exon20) | *ARAF*(synonymous ) & *NOTCH1*(intronic) | Surgical resection and ALK inhibitor | Neg | AWD/21mo; Response to ALK inhibitor/16mo |
|  |  |  | Right Thigh | Metastastic | 2 |  |  |  |  |  |  | Neg |  |
|  |  |  | Ilium | Metastastic | 2 |  |  |  |  |  |  | Neg |  |
| *Among the IMTs diagnosed in our institution from January 2016 to July 2021, as well as we collected, only these two cases displayed distant metastasis. AWD, available with disease; DOD, dead of disease; F, female; M, male; mo, month; NA, not available; Neg, negative; y, years. | | | | | | | | | | | | | |
