# Supplementary material for: Case Report: Early Distant Metastatic Inflammatory Myofibroblastic Tumor Harboring EML4-ALK Fusion Gene: Study of Two Typical Cases and Review of Literature
Source: Front Med (Lausanne). 2022 Feb 24;9:826705. doi: 10.3389/fmed.2022.826705 (PMC8907662; doi:10.3389/fmed.2022.826705)
Supplement: Supplementary file 2 [file Table_2.DOCX]

**Supplemental table 2. Previously reported metastatic inflammatory myofibroblastic tumor (IMT)**

| **Reference** | **Age(y)/ Sex** | **Location** | **Primary Tumor Size(cm)**£ | **Metastasis or invasion** | **Morphological Features of Primary Lesions** | **Morphological Features of Metastatic Lesions** | **ALK IHC** | **ALK FISH** | **Fusion partner** | **Metastatic Interval** | **Therapy** | **Prognosis** |
| --- | --- | --- | --- | --- | --- | --- | --- | --- | --- | --- | --- | --- |
| Coffin CM, et al.6* | 10/M | Lung (left) | 7.5 | Lung (right), chest wall | Three metastatic cases had classic histologic features and 3 had  atypical histologic featuresƫ | NA | NA | NA | NA | 1y | Surgery | NA |
|  | 14/F | Lung (right) | NA | Lung (left) |  |  |  |  |  | At presentation |  |  |
|  | 14/M | Lung | 14 | Brain |  |  |  |  |  | At presentation |  |  |
|  | 14/F | Retroperitoneum | 22 | Liver |  |  |  |  |  | At presentation |  |  |
|  | 16/F | Mesentery | 8 | Brain |  |  |  |  |  | 4mo |  |  |
|  | 5/F | Abdomen | NA | Lung |  |  |  |  |  | 9y |  |  |
| Debelenko, L. V. et al.7* | 10/M | posterior cervical soft tissue | 10.5 | Lung | Large vesicular nuclei with prominent nucleoli, and focally resembled ganglion cells | Microscopic foci of tumor necrosis and bronchial and vascular invasion | Pos | Neg | *CARS-ALK* | 5y | Surgery+Radiotherapy | NED/12mo |
| Gaudichon, J. et al.8* | 16/F | Left shoulder | NA | left clavicle, the arm, and the anterior chest wall soft tissues; Liver and adrenal gland | Elongated spindle cells with amphophilic cytoplasm，enlarged and moderately atypical nuclei | NA | Pos | Pos | *EML4-ALK* (FISH) | 45mo | Surgery + Crizotinib | Response /36mo |
| Telugu, R. B.et al.9* | 3/F | Suprarenal | 15 | Liver and Lung | Epithelioid morphology | NA | Pos | NA | NA | 8mo | Surgery+Chemotherapy | DOD/8mo |
|  | 13/M | Pelvic mass, omentum, and bladder | 11 | Pelvic mass, omentum, and bladder | No special | NA | Pos | NA | NA | NA | Surgery+Chemotherapy | NED/7mo |
| Jiang, Y. H.et al.10* | 37/M | Lung | NA | Bone | Histological atypicality | NA | Neg | NA | NA | NA | Surgery | DOD/15mo |
|  | 37/M | Lung |  | Liver |  |  | Neg | NA | NA |  | Surgery | DOD/15mo |
|  | 56/F | Lung |  | Pleura, liver, bone,lymph nodes |  |  | Neg | NA | NA |  | Surgery+Corticosteroids | DOD/4mo |
|  | 34/F | Maxillary sinus |  | Liver |  |  | Neg | NA | NA |  | Surgery+Radiotherapy+Corticosteroids | DOD/14mo |
|  | 46/F | Maxillary sinus |  | Liver, bone,brain |  |  | Neg | NA | NA |  | Surgery+Radiotherapy+Corticosteroids | DOD/12mo |
|  | 58/F | Maxillary sinus |  | Liver, bone |  |  | Neg | NA | NA |  | Surgery+Radiotherapy+Corticosteroids | DOD/8mo |
| Borak S, et al.11* | 74/M | Lung | NA | Mediastinal lymph nodes | Hypocellular lesion + elongated spindle cell | Highly cellular + moderate nuclear pleomorphism + coarse chromatin | Pos | Pos | NA | At presentation | NA | NA |
| Sokai A et al.12* | 67/M | Lung | 4.3 | Brain, subcutaneous and liver | Atypical nuclei and mitos | NA | Neg | Pos | *EML4-ALK* | NA | Prednisolone + Lobectomy | DOD/1mo |
| Saiki M et al.13* | 26/M | Lung&left forearm | NA | Mediastinal and abdominal lymph node, and right kidney | Typical (only specimen in left forearm) | NA | Pos | Pos | EML4-ALK (by FISH) | NA | Alectinib | Response/ 4mo |
| Yuan C, et al.1† | 18/F | Lung | 4.6 | Brain | Identical to metastatic lesions | Hypercellular + focal hyalinized hypocellularity + necrosis+multinucleated giant cells + active mitosis | Pos | NA | NA | 1mo | Surgery & Crizotinib | NED/6mo |
| Chow SC, et al.2† | 27/M | Lung | 8 | Gastroesophageal Junction | Mildly atypical spindle-shaped myofibroblasts | NA | Neg | NA | NA | At presentation | Surgery | NED/18mo |
| Zorinas A, et al.3† | 43/F | Small Intestinal | NA | Left Ventricle, liver, bones, and stomach | NA | Atypical cells and nuclei + active mitosis with atypical mitoses. | Neg | NA | NA | 1y | Surgery | DOD/9mo |
| Gallego L, et al.4† | 53/F | Lung | NA | Maxillary Region | No special | No special | NA | NA | NA | 2y | Surgery | NED/20mo |
| Carillo C, et al.5† | 52/M | Lung | NA | Adrenal gland | NA | NA | NA | NA | NA | At presentation | Surgery | NED/12mo |
| Na YS, et al.6† | 76/M | Pleura and chest wall | 7.7 | Kidney | No special | No special | NA | NA | NA | At presentation | Glucocorticoid | NED/1mo |
| Inoue M, et al.7† | 16/F | Breast | 2.2 | Intracranial, lung and pancreas | Small area necrosis + minimal atypia | NA | Pos | NA | NA | At presentation | Surgery | NED/9mo |
| Hou TC, et al.8† | 57/F | Retrosternal mass | 9.5 | Neck lymph nodes | No special | Atypical cytology + hemorrhage and necrosis + more pleomorphic pattern | Neg | NA | NA | Re/8y, Me/3.5 y | Surgery | NA |
| Zhao HD, et al.9† | 56/F | Breast | 4 | Groin | No special | Mitotic figures | Pos | NA | NA | Re/3mo&7mo, Me/10mo | Surgery and radiotherapy | NA |
| Inadomi K, et al.10† | 64/M | Mesenterium | 12.5 | Para-aortic&supraclavicular regions | Mildly atypical | NA | Neg | NA | NA | At presentation | ALK-1 inhibitor | DOD/7mo |
| Morotti RA, et al.11† | 14/F | lower esophagus, stomach, and liver | NA | Lung, abdomen, and pelvis | Identical to metastatic lesions | Ganglion-like appearance and More numerous plasma cells than primary lesions | Neg | Neg | NA | 9y | Surgery+immunosuppressive therapy | NA |
| Muñoz Moya JE, et al.12† | 12/F | Retroperitoneum | NA | Psoas, diaphragm and kidneys | little to moderate cellularity | NA | Neg | NA | NA | At presentation | Antibody and hormone therapy | NA |
| Libby EK, et al.13† | 61/M | Bladder | NA | Peritoneum and large intestines | Indistinct nuclear borders + mild nuclear pleomorphism | Similar with primary lesions | Neg | NA | NA | At presentation | Surgery | DOD/3 weeks |
| Chen, M, et al.14† | 60/M | Left adrenal area | 6.7 | Rectum | No special | NA | Neg | NA | NA | NA | Surgery+Chemotherapy | NA |
| Bonvini, P, et al.15† | 19/M | Bladder | NA | Proximal osteolytic and multiple bilateral lung | Myxoid stroma with a heterogeneous inflammatory infiltrate | NA | Pos | NA | NA | NA | Surgery + Entrectinib | NED/24mo |
| Choi, E. J. et al.16† | 27/F | Breast | 3 | Lymph nodes | Spindle cell and inflammatory cells + High mitotic activity and mild cellular pleomorphism | Increased cellularity and cellular atypia of spindle cells, high mitotic activity | Pos | NA | NA | 2y | Surgery | NA |
| Dogan, M. S.et al.17† | 3/M | Kidney | 6.0 | Lung | Typical | Similar with primary lesions | Pos | NA | NA | NA | Surgery | NED/6mo |
| Duan, J. et al.18† | 57/M | Thyroid | 4.0 | Right adductor magnus | No special | No special | Pos | NA | NA | 17mo | Surgery+radiotherapy+steroid therapy | NA |
| Ernst, C. W.et al.19† | 13/M | Pelvic mass | NA | Liver | NA | NA | NA | NA | NA | NA | NA | NA |
| Fan, J. et al.20† | 37/M | Gastric | 4.5 | Lymph-node | Focal cellular atypia | NA | Pos | Pos | NA | NA | Surgery | NED/6mo |
| Koechlin, L. et al.21† | 37/F | Spleen | NA | Lymph nodes, mediastinum, the liver, and vertebral bones | High mitotic activity and focal punctuate necrosis | NA | Neg | NA | NA | At presentation and 1mo | Surgery+Chemotherapy | NA |
| Kim, S. et al.22† | 28/F | Abdominopelvic | 8.0 | Vertebrae and liver | No special | No special | Pos | NA | NA | 14mo | Surgery+Immunosuppression and hormone therapy | NA |
| LaVigne, A. W.et al.23† | 23/M | Oral cavity | 1.3 | Regional lymph nodes | No special | NA | Pos | Pos | NA | NA | Surgery+radiotherapy+ALK-inhibition therapy | DOD/9mo |
| Liu, Q.et al.24† | 59/M | Lung | 3.7 | Bone and abdominal cavity | No special | Similar with primary lesions | Neg | NA | NA | 8mo | Surgery+Apatinib | NED/18mo |
| Lu, J.et al.25† | 20/M | Hepatobiliary and Pancreatic | NA | Hilar lymph node | No special | NA | Pos | NA | NA | At presentation | Surgery | NA |
| Moon, C. H.et al.26† | 47/M | Lung | 4.3 | Bone | No special | NA | Pos | NA | NA | 2y | Surgery+Radiotherapy | DOD/3mo |
| Song, Z.et al.27† | 55/F | Pulmonary Trunk | 3.6 | Mediastinal Lymph Node | NA | NA | NA | NA | NA | At presentation | Surgery | NA |
| Watanabe, K.et al.28† | 70/F | Iliac bone | 7 | Lung | Oval or elongated nuclei with mild pleomorphism | NA | NA | NA | NA | 1y | Surgery | DOD/24mo |
| Shimodaira, Y.et al.29† | 81/M | Rectum | 2.2 | Liver | Mild to moderate nuclear atypia | Similar with primary lesions | Neg | Neg | NA | 2mo | Surgery | DOD/1mo |
| Petridis, A. K.et al.30† | 29/M | Lung | NA | Brain | NA | NA | NA | NA | NA | At presentation | Surgery | DOD/NA |
| Jacob SV et al 31† | 45/F | Liver, adrenal gland, and pancreas | 7.0 | Lumbar spine | No special | No special | Pos | Pos | NA | At presentation | ALK inhibitor Crizotinib | NED/27mo |
| An aggressive IMT subtype EIMS was excepted，* References showing in the text; † References showing in the supplemental files; £ Including image showing；ƫ atypical histologic features: These atypical features included hypercellularity, a prominent fascicular architecture, a focal herringbone pattern, necrosis, abundant large ganglionlike cells, multinucleated or anaplastic giant cells, cellular and nuclear pleomorphism, atypical mitoses, a round or polygonal cell component, and necrosis. DOD, dead of disease; F, female; M, male; mo, Me, metastasis; month; NA, not available; NED, no evidence of disease; Neg, negative; Pos, positive; Re, recurrence; y, years. | | | | | | | | | | | | |

**Supplemental Reference**

1.Yuan C, Ma MJ, Parker JV, Mekhail TM. Metastatic Anaplastic Lymphoma Kinase-1 (ALK-1)-Rearranged Inflammatory Myofibroblastic Sarcoma to the Brain with Leptomeningeal Involvement: Favorable Response to Serial ALK Inhibitors: A Case Report. Am J Case Rep. 2017 Jul 17;18:799-804.

2.Chow SC, Nahal A, Mayrand S, Ferri LE. Pulmonary inflammatory myofibroblastic tumor invading the gastroesophageal junction. Ann Thorac Surg. 2010 May;89(5):1659-61.

3.Zorinas A, Austys D, Janusauskas V, et al. Small Intestinal Inflammatory Myofibroblastic Metastasis in the Left Ventricle. Ann Thorac Surg. 2017 Jan;103(1):e31-e33.

4.Gallego L, Santamarta TR, Blanco V, et al. Inflammatory myofibroblastic tumor of the lung and the maxillary region: a benign lesion with aggressive behavior. Case Rep Dent. 2013;2013:879792.

5.Carillo C, Anile M, De Giacomo T, Venuta F. Bilateral simultaneous inflammatory myofibroblastic tumor of the lung with distant metastatic spread. Interact Cardiovasc Thorac Surg. 2011 Aug;13(2):246-7.

6. Na YS, Park SG. Inflammatory myofibroblastic tumor of the pleura with adjacent chest wall invasion and metastasis to the kidney: a case report. J Med Case Rep. 2018 Sep 9;12(1):253.

7.Inoue M1, Ohta T, Shioya H, et al. Inflammatory myofibroblastic tumors of the breast with simultaneous intracranial, lung, and pancreas involvement: ultrasonographic findings and a review of the literature. J Med Ultrason (2001). 2018 Apr;45(2):331-335.

8.Hou TC, Wu PS, Huang WY, et al. Over expression of CDK4 and MDM2 in a patient with recurrent ALK-negative mediastinal inflammatory myofibroblastic tumor: A case report. Medicine (Baltimore). 2020;99(12):e19577.

9. Zhao HD, Wu T, Wang JQ, et al. Primary inflammatory myofibroblastic tumor of the breast with rapid recurrence and metastasis: A case report. Oncol Lett. 2013 Jan;5(1):97-100.

10. Inadomi K, Kumagai H, Takayoshi K, et al. Successful combination chemotherapy for metastatic inflammatory myofibroblastic tumor: A case report. Oncol Lett. 2015 Nov;10(5):2981-2985.

11.Morotti RA, Legman MD, Kerkar N, et al. Pediatric inflammatory myofibroblastic tumor with late metastasis to the lung: case report and review of the literature. Pediatr Dev Pathol. 2005 Mar-Apr;8(2):224-9.

12. Muñoz Moya JE, Alfaro Aguirre MO, Leiva Silva M, et al. Inflammatory myofibroblastic tumor: Variable presentation of the same pathology. Rev Chil Pediatr. 2019 Jun;90(3):328-335.

13. Libby EK, Ellis LT, Weinstein S, et al. Metastatic inflammatory myofibroblastic tumor of the bladder. Urol Case Rep. 2018 Nov 15;23:10-12.

14.Chen M, Zhang L, Cao G, Zhu W, Chen X, Fang Q. Partial response to chemotherapy in a patient with retroperitoneal inflammatory myofibroblastic tumor. Mol Clin Oncol. 2016 Oct;5(4):463-466.

15.Bonvini P, Rossi E, Zin A, Manicone M, Vidotto R, Facchinetti A, Tombolan L, Affinita MC, Santoro L, Zamarchi R, Bisogno G. Case Report: Circulating Tumor Cells as a Response Biomarker in ALK-Positive Metastatic Inflammatory Myofibroblastic Tumor. Front Pediatr. 2021 Apr 29;9:652583.

16.Choi EJ, Jin GY, Chung MJ, Moon WS, Youn HJ. Primary Inflammatory Myofibroblastic Tumors of the Breast with Metastasis: Radiographic and Histopathologic Predictive Factors. J Breast Cancer. 2015 Jun;18(2):200-5.

17.Dogan MS, Doganay S, Koc G, Gorkem SB, Unal E, Ozturk F, Coskun A. Inflammatory Myofibroblastic Tumor of the Kidney and Bilateral Lung Nodules in a Child Mimicking Wilms Tumor With Lung Metastases. J Pediatr Hematol Oncol. 2015 Aug;37(6):e390-3.

18.Duan J, Wang Y. A case report of recurrent thyroid inflammatory myofibroblastic tumor and its metastasis in soft tissue. Medicine (Baltimore). 2017 Nov;96(45):e8485.

19.Ernst CW, Van Der Werff Ten Bosch J, Desprechins B, de Mey J, De Maeseneer M. Malignant transformation of an abdominal inflammatory myofibroblastic tumor with distant metastases in a child. JBR-BTR. 2011 Mar-Apr;94(2):78-80.

20.Fan J, Huang B, Yang X, Yang M, He J, Nie X. ALK-positive gastric inflammatory myofibroblastic tumor in an adult with familial adenomatous polyposis and diffuse fundic polyposis. Diagn Pathol. 2017 Sep 18;12(1):68.

21.Koechlin L, Zettl A, Koeberle D, von Flüe M, Bolli M. Metastatic Inflammatory Myofibroblastic Tumor of the Spleen: A Case Report and Review of the Literature. Case Rep Surg. 2016;2016:8593242.

22.Kim S, Bakkum-Gamez JN, Okuno S, Kerr S, Dowdy SC. Abdominopelvic inflammatory myofibroblastic tumor that metastasized to the vertebrae and liver: A case report and review of the literature. Gynecol Oncol Rep. 2015 Feb 7;12:9-12.

23.LaVigne AW, Meredith DM, D'Adamo DR, Margalit DN. Treatment-refractory ALK-positive inflammatory myofibroblastic tumour of the oral cavity. BMJ Case Rep. 2018 Apr 11;2018:bcr2017221553.

24.Liu Q, Wei J, Liu X, Wang J. Anaplastic lymphoma kinase-negative pulmonary inflammatory myofibroblastic tumor with multiple metastases and its treatment by Apatinib: A case report. Medicine (Baltimore). 2019 Dec;98(52):e18414.

25.Lu J, Xiong XZ, Cheng NS. Hepatobiliary and Pancreatic: Inflammatory myofibroblastic tumor of the liver mimicking intrahepatic cholangiocarcinoma with hilar lymph node metastasis. J Gastroenterol Hepatol. 2019 Feb;34(2):312.

26.Moon CH, Yoon JH, Kang GW, Lee SH, Baek JS, Kim SY, Kim HR, Kim CH. A case of recurrent pulmonary inflammatory myofibroblastic tumor with aggressive metastasis after complete resection. Tuberc Respir Dis (Seoul). 2013 Oct;75(4):165-9.

27.Song Z, Zhang Y, Xu X, Li F, Shi T, Li Z, Zhou Q. Inflammatory myofibroblastic tumor invading pulmonary trunk with multiple mediastinal lymph node metastasis. Ann Thorac Surg. 2011 Jul;92(1):e15.

28.Watanabe K, Tajino T, Sekiguchi M, Suzuki T. Inflammatory myofibroblastic tumor (inflammatory fibrosarcoma) of the bone. Arch Pathol Lab Med. 2000 Oct;124(10):1514-7.

29.Shimodaira Y, Sugawara K, Fukuda S, Suzuki Y, Watanabe N, Koizumi S, Ohba R, Hiroshima Y, Matsuhashi T, Nanjo H, Iijima K. Aggressive Inflammatory Myofibroblastic Tumor without Anaplastic Lymphoma Kinase Gene Rearrangement in the Rectum with Liver Metastasis. Intern Med. 2020 Feb 15;59(4):495-499.

30.Petridis AK, Hempelmann RG, Hugo HH, Eichmann T, Mehdorn HM. Metastatic low-grade inflammatory myofibroblastic tumor (IMT) in the central nervous system of a 29-year-old male patient. Clin Neuropathol. 2004 Jul-Aug;23(4):158-66.

31.Jacob SV, Reith JD, Kojima AY, et al. An Unusual Case of Systemic Inflammatory Myofibroblastic Tumor with Successful Treatment with ALK-Inhibitor. Case Rep Pathol. 2014;2014:470340.
