# Supplementary material for: Case Report: Early Distant Metastatic Inflammatory Myofibroblastic Tumor Harboring EML4-ALK Fusion Gene: Study of Two Typical Cases and Review of Literature
Source: Front Med (Lausanne). 2022 Feb 24;9:826705. doi: 10.3389/fmed.2022.826705 (PMC8907662; doi:10.3389/fmed.2022.826705)
Supplement: Supplementary file 3 [file Table_3.DOCX]

**Supplemental table 3. Previously reported inflammatory myofibroblastic tumor with *EML4-ALK* fusion**

| **Reference** | **Age/ Sex** | **Location** | **Mestasis** | **Size (cm)** | **Pathological features** | **ALK IHC** | **ALK FISH** | **Fusion gene** | **Therapy** | **Prognosis** |
| --- | --- | --- | --- | --- | --- | --- | --- | --- | --- | --- |
| Gaudichon, J. et al.^8*^ | 16/F | Left shoulder | Left clavicle, the arm, and the anterior chest wall soft tissues; Liver and adrenal gland | NA | Elongated spindle cells with amphophilic cytoplasm，enlarged and moderately atypical nuclei | Pos | Pos | EML4-ALK | Surgery + Crizotinib | Response /3y |
| Sokai A *et al.*^12*^ | 67/M | Lung | Brain, subcutaneous and liver | 4.3 | Atypical nuclei and mitos | Neg | Pos | EML4-ALK | Prednisolone + Lobectomy | DOD/1mo |
| Saiki M et al.^13*^ | 26/M | Lung&left forearm | Mediastinal and abdominal lymph node, and right kidney | NA | Typical (only specimen in left forearm) | Pos | Pos | EML4-ALK (by FISH) | Alectinib | Response/4mo |
| Antonescu CR *et al.*^14*^ | 6mo/F | Arm | NA | NA | Typical | Pos | Pos | EML4 exon 4 & ALK exon 20 | NA | NA |
|  | 6/F | Lung | NA |  |  | Pos | Pos | Unknown |  |  |
|  | 5/M | Lung | NA |  |  | Pos | NA | Unknown |  |  |
|  | NB | Omentum | NA |  |  | Pos | NA | Unknown |  |  |
|  | 36/F | Trachea | NA |  |  | Pos | NA | Unknown |  |  |
|  | 18/F | Lung | NA |  |  | Neg | NA | Unknown |  |  |
|  | 39/F | Lung | NA |  |  | Pos | NA | Unknown |  |  |
| Muscarella LA *et al.*^15*^ | 74/F | hypopharynx | NA | 5 | Spindle‑to‑epithelioid cells proliferation + active mitosis | Pos | Pos | EML4 exon 6 &ALK exon 20 | Surgery without ALK inhibitors | DOD/11mo |
| Jiang Q *et al.*^16*^ | 45/M | Abdominopelvic cavity | Intraperitoneal invasion | 5 | Nuclear atypia and mitotic figures. | Pos | Pos | EML4 exon 6 & ALK exon 20 | Surgery and Crizotinib | Could not tolerate ALK inhibitors and eventually died of organ failure. |
| Vargas-Madueno F *et al.*^17*^ | 61/F | Lung | NO | 3 | Typical | Pos | Pos | EML4 exon 2 & ALK exon 20 | lumpectomy, chemotherapy, and radiation | NED/12mo |
| Chang, J. C.et al.^18*^ | 41/F | Left lung | NA | NA | Typical | Pos | Neg | EML4-ALK | NA | NA |
|  | 40/F | Right lung | NA | 2.6 |  | Pos | Neg | EML4-ALK |  |  |
| Lopez-Nunez, O.et al.^19*^ | Infant | Head | NO | 5 | Hypocellular pattern+glomeruloid-like feature | Pos | Pos | EML4-ALK | Surgery + Crizotinib | NED/17mo |
| DOD, dead of disease; F, female; M, male; mo, month; NA, not available; NED, no evidence of disease; Neg, negative; Pos, positive; y, years. | | | | | | | | | | |
