# Supplementary material for: Case Report: Early Distant Metastatic Inflammatory Myofibroblastic Tumor Harboring EML4-ALK Fusion Gene: Study of Two Typical Cases and Review of Literature
Source: Front Med (Lausanne). 2022 Feb 24;9:826705. doi: 10.3389/fmed.2022.826705 (PMC8907662; doi:10.3389/fmed.2022.826705)
Supplement: Supplementary file 4 [file Data_Sheet_1.docx]

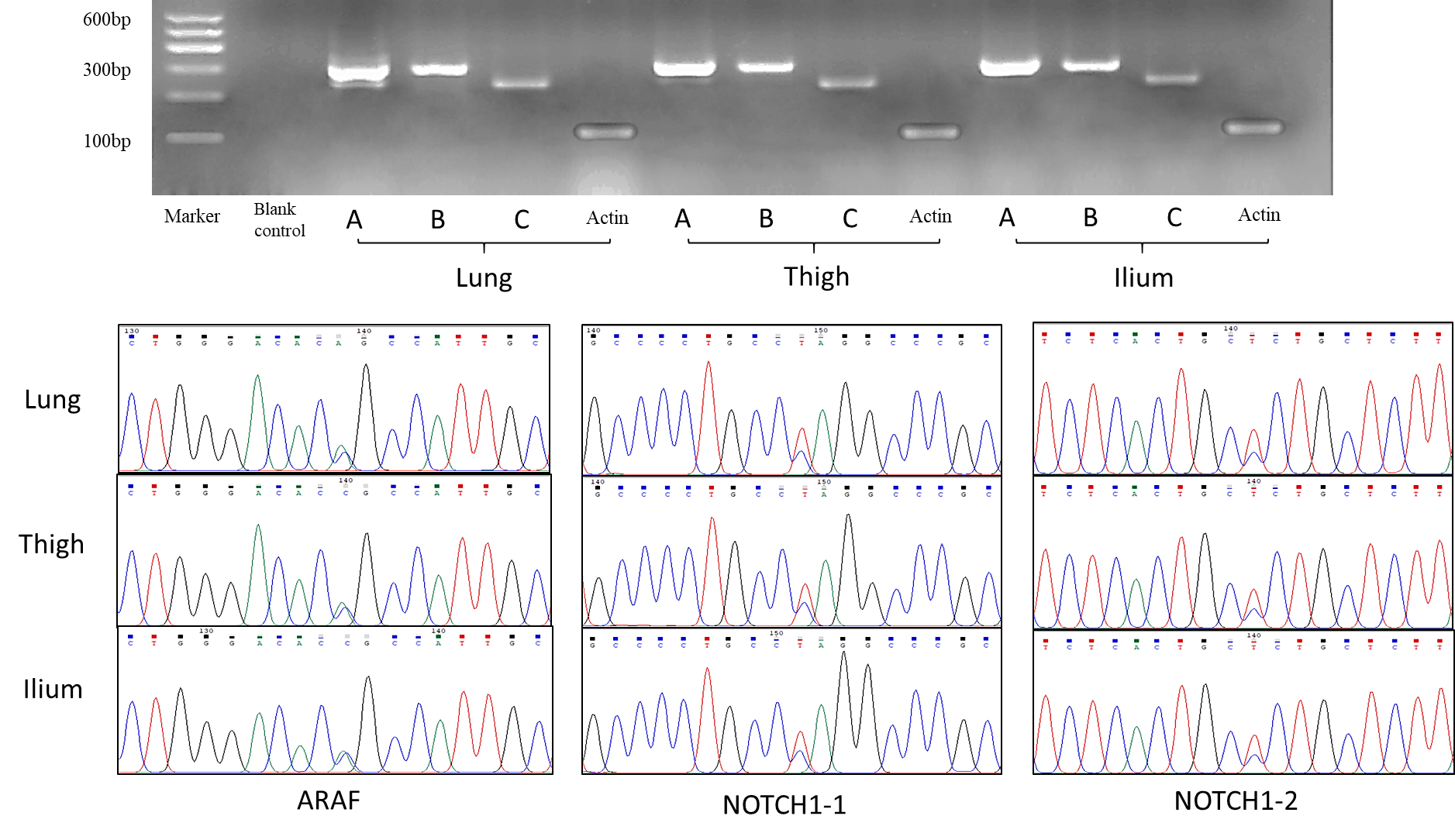


Supplemental Fig.1. The synonymous variant of *ARAF* and intron variant of *NOTCH1* were detected in the lung, thigh and ilium lesions simultaneously.

Mutation of *ARAF* is c.228A>C(p.Thr76Thr) in chrome X. Mutation of *NOTCH1*-1 is c.62-3T>C in Chrome 9; mutation of *NOTCH1*-2 is c.3902-17C>T in Chrome 9. A, B, C represents the gene of *ARAF*, *NOTCH1*-1 and *NOTCH1*-2 respectively.
