# Supplementary material for: Case Report: Early Distant Metastatic Inflammatory Myofibroblastic Tumor Harboring EML4-ALK Fusion Gene: Study of Two Typical Cases and Review of Literature
Source: Front Med (Lausanne). 2022 Feb 24;9:826705. doi: 10.3389/fmed.2022.826705 (PMC8907662; doi:10.3389/fmed.2022.826705)
Supplement: Supplementary file 5 [file Data_Sheet_2.DOCX]

**Supplemental Files for Methods**

1. **Antibodies performed and subcellular distribution**

| **Antibody** | **Laboratory** | **Clone** | **Dilution** | **Subcellular distribution** |
| --- | --- | --- | --- | --- |
| ALK | Dako, Carpintera CA | clone ALK1 | 1:20 | Cytoplasm/Nucleus |
| SMA | Dako, Carpintera CA | clone 1A4 | 1:100 | Cytoplasm/Nucleus |
| Desmin | Dako, Carpintera CA | clone D33 | 1:100 | Cytoplasm |
| Cytokeratin | Dako, Carpintera CA | monoclonal AE1/AE3 | 1:200 | Cytoplasm/Membrane |
| EMA | Dako, Carpintera CA | monoclonal E29 | 1:100 | Cytoplasm/Membrane |
| Myogenin | ZSGB-Bio | clone F5D | 1:50 | Cytoplasm |
| CD34 | Buffalo Grove, IL | Buffalo Grove, IL | 1:50 | Cytoplasm |
| S-100 protein | Dako, Carpintera CA | polyclonal anti-S-100 | 1:400 | Cytoplasm |
| Ki-67 | Dako, Carpintera CA | monoclonal MIB-1 | 1:100 | Nucleus |

| AKT1 | ALK | ARAF | ATM | BIM | BRAF | BRCA1 | BRCA2 | CCND1 | CDK4 |
| --- | --- | --- | --- | --- | --- | --- | --- | --- | --- |
| CDK6 | CDKN2A | CYP2C19 | CYP2D6 | CYP3A4 | DDR2 | DPYD | EGFR | ERBB2 | ERBB3 |
| ERBB4 | FGF19 | FGF3 | FGF4 | FGFR1 | FGFR2 | FGFR3 | FLT3 | HRAS | JAK1 |
| JAK2 | KDR | KIT | KRAS | MAP2K1 | MET | MTOR | NF1 | NRAS | NRG1 |
| NTRK1 | NTRK2 | NTRK3 | PDGFRA | PIK3CA | PTCH1 | PTEN | RAF1 | RET | ROS1 |
| SMO | STK11 | TP53 | TSC1 | TSC2 | UGT1A1 |  |  |  |  |

1. **56 gene panel for NGS**
2. **Primer for case one**

**The primer for *EML4-ALK* fusion**

**Forward:** EML4: 5’-ACTGATGGAG GAGGTCTTGC-3’; **Reverse**: ALK: 5’-ACCAAAACTGCAGA CAAGCA-3’.**^[1]^**

1. **Primer for case two**
2. **The primers for *EML4-ALK* ^[1]^**

| Primer | Forward | Reverse |
| --- | --- | --- |
| Primer 1 | EML4: CAAAGCA GTAGTTGGGGTTG | ALK: ACCAAAACTGCAGA CAAGCA |
| Primer 2 | EML4: GTCTTGCCAGCAAAG CAGT | ALK: ACCAAAACTGCAGA CAAGCA |
| Primer 3 | EML4: ACTGATGGAG GAGGTCTTGC | ALK: ACCAAAACTGCAGA CAAGCA |

1. **The primer for NOTCH1 and ARAF**

| Number | Gene | Forward primer（5’-3’） | Reverse primer (5’-3’） | Exon |
| --- | --- | --- | --- | --- |
| 1 | ARAF | 5'-AATTTCTAGAGTTGCTGTGATGAGA -3' | 5 ´-GGGAAGTTTGAGACCTGGTCAAG-3' | Exon 4 |
| 2 | NOTCH1 | 5'-TTCATGGAGGGGCTTCCTG-3' | 5 ´-TGCCCTCGACAAAGCAACAGGT-3' | Exon 2 |
| 3 | NOTCH1 | 5'-TGTGAGGGGGATGTCAACGAGT -3' | 5 ´-CATTCTTGCAGGGCTTGCCTTT-3' | Exon 24 |

1. Jiang Q, Tong HX, Hou YY, Zhang Y, Li JL, Zhou YH, Xu J, Wang JY, Lu WQ. Identification of EML4-ALK as an alternative fusion gene in epithelioid inflammatory myofibroblastic sarcoma. Orphanet J Rare Dis. 2017 May 23;12(1):97.
